# Assessing Concordance of Results: A Comparative Study of the Manual and Automated Urinalysis Methods

**DOI:** 10.1155/2024/6963423

**Published:** 2024-04-20

**Authors:** Nicholas Kwame Afriyie Gyamfi, George Nkrumah Osei, Ruth C. Brenyah, Lawrence Duah Agyemang, Paulina Ampomah, Kwame Osei Darkwah, Emmanuel Toboh, Richard K D Ephraim

**Affiliations:** ^1^Department of Medical Laboratory Science, School of Allied Health Sciences, University of Cape Coast, Cape Coast, Ghana; ^2^Department of Clinical Microbiology, School of Medical Sciences, Kwame Nkrumah University of Science and Technology, Kumasi, Ghana; ^3^Public Health Unit, Komfo Anokye Teaching Hospital, Kumasi, Ghana; ^4^Department of Biomedical Sciences, School of Allied Health Sciences, University of Cape Coast, Cape Coast, Ghana; ^5^College of Natural Sciences, Institute of Molecular Biology and Genetics, Jeonbuk National University, Jeonju, Republic of Korea; ^6^Laboratory Unit, Dansoman Polyclinic, Accra, Ghana

## Abstract

**Introduction:**

An accurate urine analysis is a good indicator of the status of the renal and genitourinary system. However, limited studies have been done on comparing the diagnostic performance of the fully automated analyser and manual urinalysis especially in Ghana. This study evaluated the concordance of results of the fully automated urine analyser (Sysmex UN series) and the manual method urinalysis at the Komfo Anokye Teaching Hospital in Kumasi, Ghana. *Methodology*. Sixty-seven (67) freshly voided urine samples were analysed by the automated urine analyser Sysmex UN series and by manual examination at Komfo Anokye Teaching Hospital, Ghana. Kappa and Bland-Altman plot analyses were used to evaluate the degree of concordance and correlation of both methods, respectively.

**Results:**

Substantial (*κ* = 0.711, *p* < 0.01), slight (*κ* = 0.193, *p* = 0.004), and slight (*κ* = 0.109, *p* < 0.001) agreements were found for urine colour, appearance, and pH, respectively, between the manual and automated methods. A strong and significant correlation (*r* = 0.593, *p* < 0.001) was found between both methods for specific gravity with a strong positive linear correlation observed for red blood cell count (*r* = 0.951, *R*^2^ = 0.904, *p* < 0.001), white blood cell count (*r* = 0.907, *R*^2^ = 0.822, *p* < 0.001), and epithelial cell count (*r* = 0.729, *R*^2^ = 0.532, *p* < 0.001). A perfect agreement of urine chemistry results in both methods was observed for nitrite 67 (100%) (*κ* = 1.000, *p* < 0.001) with a fair agreement for protein 46 (68.7%) (*κ* = 0.395, *p* < 0.001). A strong agreement was found in both methods for the presence of cast 65 (97.0%) (*κ* = 0.734, *p* < 0.001) with no concordance observed for the presence of crystals (*κ* = 0.115, *p* = 0.326) and yeast-like cells (YLC) (*κ* = 0.171, *p* = 0.116).

**Conclusion:**

The automated and manual methods showed similar performances and good correlation, especially for physical and chemical examination. However, manual microscopy remains necessary to classify urine sediments, particularly for bacteria and yeast-like cells. Future research with larger samples could help validate automated urinalysis for wider clinical use and identify areas requiring improved automated detection capabilities.

## 1. Introduction

Urinalysis, one of the most routinely performed clinical tests, provides significant information for early screening, monitoring, and prognosis of kidney and urogenital tract disorders as well as other metabolic conditions [1–6]. General indications for urinalysis include the possibility of glycosuria, proteinuria, ketosis, or acidosis/alkalosis in pregnant women and patients with diabetes mellitus or metabolic states; stone formation or urinary tract infection; non-infectious renal disease secondary to systemic diseases or to the adverse effects of drugs; and noninfectious post-renal disease [7, 8]. The urinalysis test involves an initial assessment of the physical and chemical characteristics of urine followed by urine sediment analysis [1, 8–10].

The manual method includes a visual inspection for urine colour and appearance, a dipstick test, and a microscopic examination of the urine sediment [10]. The manual urine dipstick analysis is however subjective to the colour interpretation of the observer and as such has a higher chance of giving false positive or negative results [1]. Similarly, the manual microscopic analysis is tedious to perform, time demanding, has higher interobserver variability in the urine particle counting, and hence requires well-trained and experienced staff making it less suitable to be used in routine practice [3, 7, 10–12]. Also, procedures such as sedimentation and decantation of urine samples in the preanalytic phase of manual microscopic analysis may lead to cell lysis and loss of formed elements, thus resulting in false negative results [11, 13]. Therefore, the automated urine analysers were developed to provide better standardization, improve the certainty of measurement, and save staff time [1, 3, 7, 14].

However, laboratories that have made the transition from manual microscopic methods to automatic systems still have some concerns about the concordance of results generated from both methods. Limited studies have been done on comparing the diagnostic performance of the fully automated analyser and manual urinalysis in West and sub-Saharan Africa, particularly Ghana. This study evaluated the concordance of results of the fully automated urine analyser and the manual method of urinalysis at Komfo Anokye Teaching Hospital, Ghana. The findings of this study will help present the correlation of the fully automated urine analyser with the manual method to guide decision-making in the procurement and use of automated urinalysis equipment to support the diagnosis.

## 2. Methodology

### 2.1. Study Site, Duration, Design, and Population

A comparative study was conducted at the Komfo Anokye Teaching Hospital (KATH), Ghana. The study period was from June 2022 to September 2022. A simple random sampling technique was employed to select urine samples received in the Parasitology Laboratory of the hospital. A total of 67 urine samples were used for the study.

### 2.2. Eligibility Criteria

Ten (10) ml of freshly voided midstream urine samples in a sterile container that had not exceeded 1 hour upon collection were eligible for the study. Urine samples of volume less than 10 ml and those that had stood for more than 1 hour were excluded. Also, urine samples that were known to contain preservatives were excluded.

### 2.3. Ethical Consideration

The study was approved by the Department of Medical Laboratory Science, University of Cape Coast. This student project was performed in accordance with the Helsinki Protocols on research ethics. All methods were carried out following relevant guidelines and regulations. Confidentiality was also observed throughout the study. All samples were anonymized by labelling with new numbers that had no link to the patient identification to reaffirm anonymity.

### 2.4. Sampling Collection Procedure

All urine samples received were analysed within 1 hour of collection. Each urine sample was mixed thoroughly and divided into two aliquots; one was analysed manually and the other by the fully automated Sysmex UN series urine analyser. The physical, chemical, and microscopic components of each urine sample were analysed. The collection, preparation of specimens, and urinalysis were performed according to European Urinalysis Guidelines [8].

#### 2.4.1. Manual Urine Analysis

The physical properties (colour and appearance) of the urine samples were first observed macroscopically. Urine samples were then analysed with a urine dipstick for their chemistry parameters. Samples were then centrifuged at 1500 rpm (400 g) for 5 minutes and decanted until about 0.5 ml of urine remained at the bottom of the tube. The sediment was resuspended, after which one drop of sediment was placed on a glass slide, covered with a 20 × 20 mm coverslip, and examined under a microscope [7]. A minimum of 10 fields at 400x and 100x magnification was examined. The counts were given as an average per field (per low-power field (LPF) and per high-power field (HPF)). To minimize interobserver variability, all manual microscopic examination was performed by one qualified medical laboratory scientist (MLS) and reviewed by another qualified MLS for confirmation without the knowledge of the initial results.

#### 2.4.2. Automated Analysis

The automated urine analysis was performed using the Sysmex UN series fully automated urine analyser. Quality control was performed each day. About 5 ml of the selected urine samples were transferred into 10 ml urine sample tubes, held in Sysmex 10 tube racks, and first analysed for the physical and chemical characteristics of the urine after turning the mode to normal analysis series. The sampler analysis mode was then used for the microscopic analysis of urine [15].

#### 2.4.3. The Sysmex UN Series Fully Automated Urine Analyser

The Sysmex UN series fully automated urine analyser is a new-generation urine analyser developed by Sysmex Corporation (Kobe, Japan). It is a modular system that integrates three main modules: UC-3500 (physical and chemical analyser), UF-4000 (particle analyser), and UD 10 (digital particle screening device). Each module can be used as a standalone urine analyser or integrated as a complete automated urine work area. The Sysmex UC-3500 is a urine test strip analyser that employs reflectance photometry, refractometry, and spectrophotometry to analyse urine chemical and physical properties. The UF-4000 operates based on the fluorescent flow cytometry principle to identify, classify, and quantify urine particles. The classification and quantification of urine particles are based on the sizes, shapes, and staining features of the particles [16].

#### 2.4.4. Data Analyses

Initial entry and organization of data were done using Microsoft Excel. The data were cleaned and imported into IBM SPSS statistics version 23 for analysis. The agreement between both methods in physical and chemical examination except for specific gravity was evaluated using Cohen's kappa analysis, which assesses agreement beyond chance [17]. Specific gravity was assessed using Bland-Altman analysis evaluating the difference between methods against the average while visualizing limits of agreement and proportional bias [18]. Pearson's correlation was also used to assess the strength and direction of association between the continuous data obtained from automated and manual microscopy [19]. These established statistical tests were chosen for their appropriateness in comparing diagnostic methods and recognizing sample size limitations. All analyses were done at a 95% confidence interval, and *p* values less than or equal to 0.05 were considered statistically significant.

## 3. Results

A total of 67 urine samples were analysed in this study. A substantial agreement was found between the manual and automated results for urine colour (*k* = 0.711, *p* < 0.01) with straw (82.0%) being the most consistent urine colour (Table 1). Clear urine appearance was the most consistent appearance (64.5%) with hazy appearance being the least consistent result (12.0%). Overall, there was a slight and significant agreement between the manual and automated analysis based on urine appearance (*k* = 0.193, *p* = 0.004) (Table 1).

**Figure 1:**
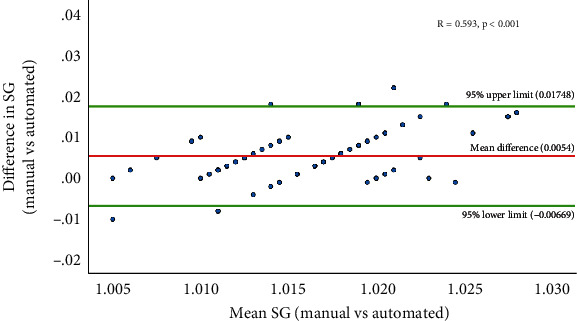
Comparative analysis of specific gravity measurements from manual and automated methods.

Table 2 shows the pairwise agreement between both methods for urine pH. A perfect concordance was found in 16 (23.9%) results with 42 (62.7%) within one grading difference. There was a slight agreement between the manual and automated methods for pH (*κ* = 0.109, *p* < 0.001) (Table 2). The mean difference between the manual and automated methods for specific gravity was 0.0054 (Figure 1). The correlation between the manual and automated comparisons for specific gravity results was strong and significant (*R* = 0.593, *p* < 0.001) (Figure 1).

**Figure 2:**
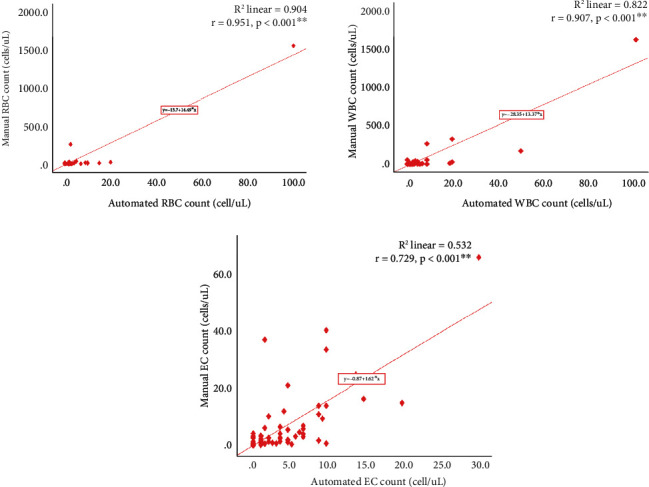
Correlation between automated and manual RBC, WBC, and EC counts. *R*^2^: coefficient of determination; *r*: Pearson correlation; RBC: red blood cell; WBC: white blood cell; EC: epithelial cell.

A perfect agreement in both methods was observed for nitrite 67 (100%) (*κ* = 1.000, *p* < 0.001) with a fair agreement seen for protein 46 (68.7%) (*κ* = 0.395, *p* < 0.001) (Table 3). A strong positive linear correlation was found between the automated and manual red blood cell (RBC) count (*r* = 0.951, *R*^2^ = 0.904, *p* < 0.001), white blood cell (WBC) count (*r* = 0.907, *R*^2^ = 0.822, *p* < 0.001), and epithelial cell (EC) counts (*r* = 0.729, *R*^2^ = 0.532, *p* < 0.001), respectively (Figure 2). The Bland-Altman plot analysis demonstrated a tendency for the manual results to be greater than the automated result in WBC and EC count as the number of WBC and EC increased in the manual count ((bias = 23.67, 95% CI = 234.13 to -186.79) and (bias = 5.43, 95% CI = 20.66 to -9.80), respectively) (Figure 2). A substantial agreement was found in both methods for the presence of cast 65 (97.0%) (*κ* = 0.734, *p* < 0.001), while a slight agreement was observed for the presence of bacteria 48 (71.6%) (*κ* = 0.065, *p* = 0.491) (Table 4). The results for crystals (*κ* = 0.115, *p* = 0.326) and yeast-like cells (YLC) (*κ* = 0.171, *p* = 0.116) showed no concordance between both methods.

## 4. Discussion

Automation of urinalysis has significantly reduced the overall workload and turnaround time but may sometimes need operator interference or manual confirmation [13]. This study evaluated the agreement between the Sysmex UN series fully automated urine analyser and manual method in terms of physical, chemical, and microscopic analyses at the Komfo Anokye Teaching Hospital, Kumasi, Ghana.

Our study revealed a substantial agreement between manual and automated analysis with regard to urine colour (*k* = 0.711, *p* < 0.01) with the most consistent result observed for straw colour (82.0%). This indicates that, in most cases, both manual and automated methods provide more consistent assessments for straw urine colour than the other urine colours. Clear urine appearance was also observed as the most consistent appearance (64.5%) with hazy appearance being the least consistent result (12.0%). This finding also stands to suggest a noticeable discrepancy between the two methods when assessing urine appearance, especially with hazy urine samples.

In dissonance with our study which found a slight agreement between the manual and automated methods in pH results (*κ* = 0.109, *p* < 0.001), Ahmed et al. found an almost perfect agreement in pH results between the two methods (*κ* = 0.914, *p* ≤ 0.001). The disparity in the results may be due to different automated analysers and sample sizes employed in both studies. Our study used a Sysmex UN series (UF-4000) analyser and a smaller sample size (67) than that employed by Ahmed et al. (sample size = 1000, H800-FUS100 automated analyser).

According to our study, a perfect agreement for nitrite 67 (100%) (*κ* = 1.000, *p* < 0.001) was observed between the manual and automated methods. This finding was consistent with Ahmed et al.'s study in 2016 which found a perfect agreement for ketone and nitrite in both methods (ketone (*κ* = 1, *p* ≤ 0.001), nitrite (*κ* = 1, *p* ≤ 0.001)) but differed from that observed by Kanegaye et al. who found a 76% agreement for nitrite in both methods [13, 20]. The disparity observed could be due to the urine samples employed in various studies. Our study and that of Ahmed et al. used voided urine, while Kanegaye et al. used catheterized urine.

This study reported a fair agreement for protein 46 (68.7%) (*κ* = 0.395, *p* < 0.001) between the two methods. This finding slightly differed from studies by Rumley and Ahmed et al. which found a 19.2% difference in protein result and a moderate agreement for protein (*κ* = 0.695, *p* ≤ 0.001), respectively [13, 21]. These findings suggest that there are minor to moderate variations between the automated analyser and the manual reading of urine dipstick tests, particularly for protein. The variations in results may be due to the subjectivity of visual test strip reading to the colour perception and interpretation of the observer which is liable to imprecision and human-related errors thereby affecting the results.

In consonance with our study which found a strong correlation between the automated and manual RBC (*r* = 0.951), WBC (*r* = 0.907), and epithelial cell (EC) counts (*r* = 0.729), Jiang et al. (RBC (*r* = 0.96), WBC (*r* = 0.98), and EC (*r* = 0.84)) and Previtali et al. (RBC (*r* = 0.98), WBC (*r* = 0.99), and EC (*r* = 0.92)) observed a strong correlation of these parameters in China and Italy, respectively [4, 22]. This indicates a high level of agreement and reliability between these two methods in assessing urinary cellular components such as RBC, WBC, and EC. Our study however observed an increased WBC and EC count in the manual than in the automated method with an increased RBC count found to be higher in the automated than manual method. This variation in results could be due to the fact that the Sysmex UN series (UF-4000) analyser employed in our study could not count slightly damaged WBC and deformed epithelial cells. The increased RBC counts obtained through the automated approach compared to the manual method could be attributed to the possibility of RBCs being lysed by the centrifugation, decantation, and resuspension in the manual method or yeast cells being counted by the automated analyser as RBCs. This finding was consistent with studies by Chien et al. and Budak and Huysal which found higher RBC and WBC cell counts with the automated analyser than the manual method [3, 6]. Moreover, the decreased number of epithelial and white blood cells produced by the automated method may underestimate the true severity of conditions compared to a manual method which could result in conditions being undertreated based on automated results alone. The higher RBC counts on automation may also overestimate blood loss through urine compared to manual differentials which may as well lead to inadvertent medical treatment or intervention.

In contrast with our study which found a substantial agreement for the presence of cast 65 (97.0%) (*κ* = 0.734, *p* < 0.001) and no concordance found for crystals (*κ* = 0.115, *p* = 0.326) and yeast-like cells (YLC) (*κ* = 0.171, *p* = 0.116) between both methods, Ince et al. found no concordance for the presence of cast ((*κ* = 0.13, *p* = 0.051) and (*κ* = 0.10, *p* = 0.135)) and a moderate agreement for bacterial and crystals ((*κ* = 0.47, *p* ≤ 0.001), (*κ* = 0.52, *p* ≤ 0.001), (*κ* = 0.54, *p* ≤ 0.001), and (*κ* = 0.57, *p* ≤ 0.001), respectively) in all two automated results compared to the manual results in Turkey [7]. Tantisaranon et al. also found a fair to moderate agreement for cast in three automated results compared to the manual method in Thailand ((*κ* = 0.42, *p* ≤ 0.001), (*κ* = 0.38, *p* ≤ 0.001), and (*κ* = 0.62, *p* ≤ 0.001)) [10]. The disparity in these findings may be due to different automated analysers and sample sizes employed in various studies. Our study used Sysmex UN series (UF-4000) analyser and a smaller sample size (67) than that employed by Ince et al. and Tantisaranon et al. (sample size = 209 and 100, (Iris iQ200 ELITE, Dirui FUS-200) and (Cobas 6500, UN3000-111b, iRICELL 3000) analysers, respectively).

This study also found a slight agreement for the presence of bacteria 48 (71.6%) for both methods (*κ* = 0.065, *p* = 0.491) with 16 samples found to be positive for bacteria in the manual method but was not observed by the automated analyser. This increased number of discrepancies for bacteria count in both methods was also reported by Chien et al., Lamchiagdhase et al., and Alves et al. indicating a high tendency of automated analysers missing bacteria presence in urine [3, 23, 24]. This discrepancy observed for bacteria could be due to the fact that they exist in various shapes and sizes which may be more difficult for automated analysers to accurately identify them. An increased number of positive results were also observed for yeast in the automated method than manual. This was similarly observed by Chien et al., Linko et al., and Lamchiagdhase et al. demonstrating a high number of false positive yeast results generated by the automated analysers [3, 23, 25]. The discrepancy observed for yeast could also be due to inaccurate identification of nonyeast elements such as smaller RBCs as yeast cells by automated analysers thereby increasing the number of false positive results for the presence of yeast. Manual microscopic confirmation for bacteria absence and yeast presence in automated generated results is therefore warranted to help guide diagnosis and optimize treatment options.

This study had a few limitations. First, our sample size was small; therefore, results may not likely be representative of the method performance in Ghana. Additionally, our study could not evaluate the sensitivity, imprecision, and reliability of both methods.

## 5. Conclusion

Our study showed good concordance of most parameters analysed in both methods, especially with the physical and chemical characteristics. We, therefore, recommend a careful manual microscopic reevaluation of automated generated particle results in cases where result defects are suspected. In addition, proper attention should be paid to specimen collection, storage, and processing to obtain reliable results on both manual and automated urinalysis. Further research on larger and more diverse sample populations will be important to comprehensively validate the clinical accuracy and applicability of automated urinalysis for wider clinical use and identify areas requiring improved automated detection capabilities.

## Figures and Tables

**Table 1:** Agreement between manual and automated urinalysis based on urine colour and appearance.

Variable				*Κ*	*p* value
Colour	Manual				
Automated	Amber	Light amber	Straw		
Amber	**1 (25.0)**	0 (0.0)	3 (6.0)		
Light amber	0 (0.0)	**16 (72.7)**	6 (9.0)	0.711	<0.001
Straw	0 (0.0)	0 (0.0)	**41 (82.0)**		
Appearance	Manual				
Automated	Clear	Cloudy	Hazy		
Clear	**40 (64.5)**	1 (1.6)	21 (30.8)		
Cloudy	0 (0.0)	**1 (33.3)**	0 (0.0)	0.193	0.004
Hazy	0 (0.0)	1 (16.7)	**3 (12.0)**		

The data were shown as *n* (%). The bold numbers indicate samples with the same result agreement. *p* value > 0.05, Kappa is not significant and there is no agreement between methods.

**Table 2:** Comparison between manual and automated pH results.

Variable										Number (*n*)	*κ*	*p* value
pH	Automated											
Manual	5.0	5.5	6.0	6.5	7.0	7.5	8.0	8.5	9.0			
5.0	**1**	*1*	*5*	4						11		
5.5		**1**	*7*	*6*						14		
6.0	*2*		**4**	*7*	*1*	1				15		
6.5			*1*	**4**						5	0.109	<0.001
7.0			*2*		**2**					4		
7.5			1		*7*	**2**				10		
8.0					2		**1**			3		
8.5					3	*1*				4		
9.0						1				1		

Bold numbers represent cases within the same grade agreement, italic numbers represent one-grade difference, and underlined numbers represent 1.5 to 2-grade differences. *p* value > 0.05, Kappa is not significant and there is no agreement between methods.

**Table 3:** Comparison of urine chemistry results between manual and automated methods.

	Variables																							
	Automated																							
Manual	Urobilinogen	Normal	Raised	Blood	+ve	-ve	Bilirubin	+ve	-ve	Ketones	+ve	-ve	Glucose	+ve	-ve	Protein	+ve	-ve	Nitrites	+ve	-ve	Leucocytes	+ve	-ve
	Normal	**63**	2	+ve	**6**	9	+ve	**0**	2	+ve	**1**	0	+ve	**2**	1	+ve	**16**	1	+ve	**2**	0	+ve	**10**	0
	Raised	1	**1**	-ve	2	**50**	-ve	0	**65**	-ve	2	**64**	-ve	0	**64**	-ve	20	**30**	-ve	0	**65**	-ve	13	**44**

The bold numbers indicate samples with the same result agreement. Kappa analysis: urobilinogen (*κ* = 0.378, *p* = 0.002), blood in urine (*κ* = 0.434, *p* < 0.001), bilirubin (-), ketone (*κ* = 0.489, *p* < 0.001), glucose (-), protein (*κ* = 0.395, *p* < 0.001), nitrites (*κ* = 1.000, *p* < 0.001), and leucocytes (-). *p* value > 0.05, Kappa is not significant and there is no agreement between methods.

**Table 4:** Comparison of urine microscopy results from manual and automated analyser.

Variables															
Manual	Automated														
	Cast	+ve	-ve	Crystal	+ve	-ve	YLC	+ve	-ve	Mucus	+ve	-ve	Bacteria	+ve	-ve
	+ve	**3**	1	+ve	**1**	6	+ve	**4**	4	+ve	**3**	7	+ve	**2**	16
	-ve	1	**62**	-ve	3	**57**	-ve	14	**45**	-ve	3	**54**	-ve	3	**46**

The bold numbers indicate samples with the same grade agreement. Kappa analysis: cast (*κ* = 0.734, *p* < 0.001), crystal (*κ* = 0.115, *p* = 0.326), YLC (*κ* = 0.171, *p* = 0.116), mucus (*κ* = 0.296, *p* = 0.012), and bacteria (*κ* = 0.065, *p* = 0.491). YLC: yeast-like cells. *p* value > 0.05, Kappa is not significant and there is no agreement between methods.

## Data Availability

All data generated or analysed during this study are available upon request from the corresponding author.
